# Essay on the Microscope

**Published:** 1855-01

**Authors:** W. H. Dwinelle


### ARTICLE XI.

*Essay on the Microscope, read before the Society of the Alumni of American Dental Colleges,* March 2, 1854. By W. H. DWINELLE, M. D., D. D. S.

THAT was a sublime utterance which came from the lips of him, who defined life, "a moment between two eternities; the eternity of the past and the eternity of the future!" So in our corporate bodies do we stand, between two eternities, the eternity of space—the unbounded universe above, and the equally unbounded space and creation beneath.

With the telescope directing its far-seeing eye heavenward, from this stand-point the earth, the veil of mortality seems withdrawn, and the broad universe opens to the clear vision in all its beauty and sublimity. Worlds on worlds, systems upon systems, each revolving around its center, and all revolving around each other, to all eternity! The mind is overwhelmed in contemplation, and sinks within itself with emotions too deep for utterance; or if the impassioned tongue is freed to express the least of its burning, heaven-born inspiration, it is the voice of prayer, of adoration and of praise, to the infinite wisdom and goodness, who created alike each grain of sand that is swayed upon the restless ocean's shore and the myriads of worlds which roll above.

If the telescope unfolds to our view the infinitude of space above, so does the microscope open to us the infinitude of space below; showing us organization, action, life, and, for aught we know, *intelligence*, in the vast busy circulating universe revolving at our feet.

The words of Shelly, ever like those of the true poet, seem prophetic when he says :—

"How strange is human pride!
I tell thee that those viewless things,
To whom the fragile blade of grass,
That springeth in the morn,
And perisheth ere noon,
Is an unbounded world!
I tell thee that those viewless beings,
Whose mansion is the smallest particle
Of the impassive atmosphere,
Think, feel and live like man ;
That their affections and antipathies,
Like his, produce the laws,
Ruling their moral state ;
And the minutest throb,
That through their frame diffuses,
The slightest, faintest motion,
Is fixed and indispensable
As the majestic laws,
That rule yon rolling orbs."

His prophecies are corroborated by the subsequent developments of science. The true poet looks with the vision of inspiration into the future, and with his far-reaching ken anticipates the slow, plodding unfoldings of time.

If the telescope carries us above, until the mind is lost in contemplation of the vastness of space, the microscope carries us as far beneath, developing creation upon creation, until the eye is weary of peering into the domain of the infinite. The stars of heaven are imaged as deeply beneath the bosom of the ocean, as their clear ethereal home lies above it.

And thus we stand, balancing between two vast infinitudes, and this is life, from whose narrow point we look upward, into the unlimited regions of space, and from whence we look downward into the dizzy boundless depths below.

The miscroscope alike with the telescope, or rather the teachings of both, conspire to render size and space mere relative terms. The depths of space are as deep as the heights are high, and both are immeasurable. The rolling planet revolving in its orb, and the minutest grain of dust, are only *relatively* great or small, borrowing for a time, through the aid of science, the vision of the Infinite, we feel that we have but a step further to take to appreciate a condition where even *time* may cease to be relative, is unnecessary, "where it shall be no more, where eternity's beginning is forever!"

We see every thing superficially, we are blinded and traversing in the dark, amid beauties of surpassing wonder; we are deaf to the music, to the gentle cadence, the low breathing melody of heaven, which even now, is vibrating around us in harmony with the busy insect life, the rolling spheres and all of the *oneness* of God's ever vital infinite creation.

It is the province of science to lift the veil, draw aside the thick curtain that shuts out the light which illuminates the splendid glories of God's great handiwork; to quicken the senses, and make them keen to feel and faithful to transmit to the wondering soul the celestial harmonies which never had reached the dark recesses of its clay-bound abode before.

Truth is a unit, God, and all beauty and goodness and intelligence, in all of their myriad manifestations, conspire in united harmony to establish the *oneness* of truth. Truth is the diagonal mean of all of the influences and principles, and actions and laws of the universe.

All investigations and researches into the secret recesses or broad arcana of nature, ever brings back its sure reward of knowledge and truth—something more of the divine—something more of the familiar language of heaven—something more of the thoughts of angels, another instalment from God's great laboratory of knowledge, another evidence of his infinite goodness and love.

Science never established a falsehood, for it harmonizes only with truth. Science is but a comprehension and arrangement of facts, principles and truths, established from the beginning of time, though they may have been of recent discovery. So its lessons need never be feared; its proper teachings are always wholesome and profitable, refining our inner natures, elevating the soul in contemplation of the great Creator, and lifting the heart in adoration heavenward, as truly and naturally as the morning dews rise towards the sun.

There is more religion in nature, in a single leaf, a flower, a rock broken from the mountain, or a handful of dust from underneath our feet, more "evidences" of God and immortality than in all of the works on *speculative* theology ever written.

I do not wish to be understood as advocating natural theology, independent of and to the exclusion of revelation; they should go hand in hand, for they reflectively endorse and sustain each other. I condemn the blind and exclusive adherence to one as well as to the other; but when men turn their back upon nature and the sciences, and closeted from the world give themselves up entirely to speculation, they refuse to recognize many a palpable and substantial corner stone of the foundation, many a stately pillar of the superstructure, and many a gorgeous and swelling dome crowning the beautiful temple of God's holy religion.

Horace Smith, in his beautiful hymn to the flowers, says:—

> "Were I, Oh God, in churchless lands remaining,
> Far from all voice of teachers or divines,
> My soul would find in flowers of thy ordaining,
> Priests, sermons, shrines!"

I hold in my hand a fragment of Dover's cliff. It is a buried city, a representative of life in other days; a Pompeii or an Herculaneum exhumed. Here was the busy mart, there the crowded street; here the room where joy and gaiety ruled the hour, and there the *cell* where the inmate toiled its brief life, and each infusorium has left its skeleton behind.

We see every thing superficially with our unaided vision, consequently in all departments of science we were continually falling into error. We saw nature and her operations from one point of view, we caught some single manifestation to the exclusion of others, equally important to a correct uuderstanding of her principles and her actions, and where the perfection of order reigned we saw nothing but confusion. But with the aid of the microscope, we are enabled to see with a comparatively clear vision; harmony springs up out of chaos, and the manifestations of wisdom and beauty from seeming disorder.

"The term microscope, derived from two Greek words, μιχρος small and δχοπεω I view, and said to have been first suggested by Demisianus, is applied to an instrument which enables us to see distinctly and to investigate objects placed at a short distance from the eye, or to see such minute objects as without its aid would be invisible. The early history of this instrument, like that of many others of a scientific nature, is involved in considerable obscurity, so that not even the time of its discovery, nor the name of the discoverer, can be fixed on with any degree of certainty, but as in its most simple form, the microscope consisted of little or nothing else than the magnifying power or lens, which must of necessity have been made of glass or some other highly refracting material, its invention may with safety be referred to a period anterior to the christian era. Aristophanes, who lived five centuries before Christ, speaks in his *Clouds* of a burning sphere. Seneca, who was born during the first year of the christian era, and died A. D., 65, writes, that small and indistinct objects become larger and more distinct in form when seen through a globe of glass filled with water. Pliny, who died A. D. 79, mentions the burning property of lenses made of glass. Ptolemy, the celebrated astronomer of Alexandria, who flourished in the latter part of the first century, was evidently cognizant of the existence of magnifying glasses, and makes use of the word *refraction*, in his work on optics. The testimony of these ancient writers, however, is only important as proving the existence of the microscope, in its most simple and rudimentary form, viz. as an instrument composed of a simple magnifying glass or sphere, whose chief application appears to have been that of concentrating the heating power of the sun's rays.

It must have been known to the ancient Egyptians, as they have left behind them specimens of engraving now in existence, whose characters are so small that they can only be discerned by the aid of a microscope, and consequently could only have been made by the use of one. In some of the recent excavations made at Ninevah, a plano-convex lens made of rock crystal was found corresponding in its general character to those made at the present day. It is now in the British Museum.

The form of the simple microscope, or that of a single lens, was the only one that we have any definite record of until about the year 1590, when the compound microscope was invented by Zacharias Jausen, of Middleburg, in Holland.

In 1660, Robert Hooke, presented to the world his celebrated compound microscope, consisting of three lenses. He was the first to describe a method of illuminating opaque objects by means of a condenser, and was also "the first to inform us of an accurate method of finding the magnifying power of a compound microscope, than which a better plan has not been suggested in modern times;" to Hook, also belongs the merit of having "first made globule lenses of high power."

In the year 1673, the name of the immortal Leeuwenhock, became distinguished as a discoverer of numerous wonders by the aid of the microscope; his instruments, which were composed of single lenses, are said to have been greatly superior to all that had been previously made.

In 1672, Sir Isaac Newton having perfected his telescope on the *reflecting* principle, applied the same principles to the microscope, and produced the compound reflecting microscope, since so greatly improved by Arnice, Cuthbert and Dr. Goring.

About the year 1702, Mr. J. Wilson brought before the public his far-famed pocket microscope of the simple form. The instruments of Leeuwenhock and Wilson were so perfect of their kind, that for many years the unwieldy form of the compound microscope was entirely superseded by them.

In 1738, Dr. Nathaniel Lieberkuhn, of Berlin, gave a new impulse to microscopic science, by his invention of the solar microscope, which has since been brought to such a high degree of perfection. He also greatly improved the simple microscope, especially one of the pocket form, for examining opaque objects. At a later period, he produced a remarkable instrument for viewing transparent objects, with which he made discoveries in regard to physical organization and texture, which will ever make his name illustrious.

In 1740, the names of Cuff, Martin and Adams, of London, became eminent in the department of microscopic science. Cuff greatly improved the microscope of Wilson, adding a mirror to it, and seems to have been the first to suggest the present form of the instrument. In 1750, the cumbrous compound instrument then in use was greatly improved by Mr. Culpeper and Mr. Scarlet; they first employed a concave mirror for reflecting the light through the object and the compound body. From this time to 1787, the microscope in its single and compound unchromatic form was brought to great perfection. The names of the two Adams, Martin, Baker and Delabbarre, Ellis, Hill, Swarmverdam, Needham and Withering, became eminent in connection with it and the wonderful discoveries it developed during these times. These instruments continued in use until 1815, when Professor Arnici, of Modena, invented a reflecting microscope, so far superior to that of Newton, that on its being further perfected by Dr. Goring, of England, for many years it superseded every other form.

Experiments and discoveries by Sir David Brewster, Dr. Goring and Mr. Pritchard again brought the single microscope to still higher degree of perfection. At the suggestion of Sir David Brewster, Mr. Pritchard succeeded in 1824 in making the first diamond lens, which from its high refractive power, could be made of one-third the thickness of other lenses and possess the same magnifying power. In 1829, Dr. Wallarton invented his *doublet*, since so much perfected and is in use to the present day, having an angle of aperture of 35° to 50°. In 1832, Mr. Holland invented his *triplet*, which is capable of transmitting a pencil of light of 65°.

We are told that achromatism was discovered by Chestermore Hall, of Essex, England, in 1729. In 1757, Mr. Dolland, also of England, made the first perfect achromatic object glasses; his experiments were instituted from suggestions of Euler. In 1816, Frauenhofer, of Munich, further perfected the achromatic form.

In 1823, experiments were commenced in France, by Mr. Selligues, which were followed by Frauenhofer, in Munich, by Arnici, of Modena, by M. Chevalier, of Paris, and by the late Dr. Goring and Wm. Tulley, of London; all of which had for their end the improvement of achromatic object glasses.

"To M. Selligues we are indebted for the first plan of making an object glass composed of four achromatic compound lenses, each consisting of two lenses, they could be used combined or separate. A microscope constructed on this principle by M. Chevalier, was presented by M. Selligues, to the *Academie des Sciences*, in 1824. In the same year and without a knowledge of M. Selligues' success, the late Mr. Tulley, at the suggestion of Dr. Goring, constructed an achromatic object glass, for a compound microscope, composed of three lenses, which resulted in corrections throughout the field of great accuracy. He afterwards added another combination in front of these when it had an angle of aperture of 38° with a power of 300 diameters."

While these experiments were in progress, Dr. Goring made his remarkable discovery that "the penetrating power of the microscope depends upon its angle of aperture." In 1825, M. Chevalier, and in 1826, Prof. Arnici, exhibited achromatic microscopes of superior construction. About this time, "the subject of achromatism engaged the attention of some of the most profound mathematicians in England, Sir John Herschell, Professors Airy and Barlow, Mr. Coddington and others. In the year 1829, Joseph Jackson Lister, Esq., read a paper before the Royal Society, in which he made known the discovery of principles and results by which he was enabled to form a combination of lenses, capable of transmitting a pencil of 50° with a large field correct in every part. This paper, which was the ground-work of all the great improvements that have been subsequently made in the achromatic object glasses, has tended to raise the compound miscroscope from its primitive, and almost useless, condition to that of being the most important instrument ever yet bestowed by art upon the investigator of nature, and has gained for the discoverer a lasting reputation."

From this time forward, the microscope has been continually improving under the direction of numerous foreign artists, but mainly in Europe, under the hands of Powell, Ross, Smith and Chevalier.

The compound achromatic microscope has, however, absorbed almost the exclusive attention of all of the first class makers of this instrument for many years past. It is now become so highly perfected, both in the combination of its glasses and the construction of its form, as to leave but little to desire in either of these respects.

"The compound microscope differs from the simple one in having the image of an object formed by an object glass, further magnified by one or more lenses, forming an eye-glass. For a microscope to be a compound one, it is, therefore, necessary it should have objectives which form an image at a given point above them, and eye pieces with which this image is taken up, magnified again and presented to the eye." Without going into a further description of the instrument in this form, we will turn to a more pleasing part of our paper.

Pursuing the course of progress in the history of the microscope, we are now constrained to take leave of our foreign colaborers in this beautiful field of science, and travel westward to our own land, even to the forests of America, where, in an obscure village, we find a young man surrounded by various scientific works in different languages, and by implements and machinery of his own construction, engaged in a problem in optics, which has for its result the exemplification of a great truth and the denial of a proposition endorsed by, and almost a part of the creed of, the most distinguished microscopists of Europe.

At the commencement of his career he had never seen a microscope, except in its simplest form. The first lens he ever saw constructed, he made himself, nor to this day has he ever received any practical instruction from any one, yet nothing daunted, he presses forward to the sure development of a great principle which shall give another grand impulse to the physical sciences and shed a fadeless lustre upon his name.

That young man is Spencer. He has for his aim the extentension of the angle of aperture of objectives to an extent only limited by the legitimate character of a circle itself.

At the commencement of his investigations, the largest angle of aperture obtained by any optician did not exceed 70°, which, although they gave great definition, were lamentably deficient in penetrating or *resolving* power.

Leaving the imperfect deductions of theory to satisfied conservatism, and with a larger faith in the established law discovered by Dr. Goring, that "the penetrating power of a microscope depended upon its angle of aperture," he strove onward to a result, which a true recognition of that law made certain.

Qualified by a thorough study of the laws of optics, his experiments embraced the entire field of the manufacture of glass, practically testing at each stage of progress, all of their characteristics as developed by the influence of light.

"Many new, unexpected and valuable results were obtained, in reference to both the optical and physical characters of this interesting material. During the long course of labor and study, which these investigations rendered necessary, new laws of combination appeared, involving intricate theoretical investigations, and giving promise of results before unattained, not only with the new materials, but (from increased knowledge of its optical characters) with those also in common use among artists.

"To fulfil the conditions which were imposed by these new laws, in connection with those previously announced by Lister, it became a practical question how far skill in manipulation, to give the requisite curves and mechanical construction to secure most, if not all the practical conveniences required by the observer, could be made available."

Pursuing this course of inquiry, which continually resulted in new developments, at the very time that Mr. Ross, one of the first of European artists, announced that "with his $\frac{1}{12}$ objective he had obtained an angle or aperture as high as 135°, *the largest angular pencil that can pass through a microscopic object glass*," Spencer had already gone beyond this, even with his lower objectives, and shortly afterwards reached an angle of 170°, and although he did not make this large extension of angle immediately available, yet the problem of the practical ex tension of the angle to that degree, was, nevertheless, fully demonstrated to the mind of the artist. By incessant application for two years and a half, he succeeded in producing objectives as low as one-eighth of this angle, perfect in all of their corrections and of unequalled excellence.

Not satisfied with this, he persistingly pushed forward toward the ultimatum, which he considered "had no existence short of 180°," and to the surprise of every one but himself, he has recently attained an angle of 178° in his $\frac{1}{12}$ objectives, thoroughly corrected in all of their relations, and of the highest practical availability: an extraordinary result, stamping the microscope for the time, and for all time, as a perfect instrument.

As an evidence of the superiority of Spencer's objectives, it is only necessary to state, that tests which are with difficulty resolved by foreign objectives of $\frac{1}{4}$, $\frac{1}{8}$, $\frac{1}{12}$ and $\frac{1}{20}$ powers, are easily and distinctly resolved with his $\frac{1}{2}$, $\frac{1}{4}$ and $\frac{1}{8}$. In 1846, the navicula hippocampus was a severe test for an English $\frac{1}{20}$ objective; Spencer's $\frac{1}{2}$ objective readily resolves it into dots. Several other of the highest tests of that time can now be resolved with a 1 inch of Spencer's.

A committee appointed some two years ago by the American Medical Association, for the advancement of science, after thoroughly testing and comparing them with the best objectives of foreign make, decided "that his glasses were quite superior to any now made in the world."

It is a matter of pride and congratulation that America, with her Spencer, stands pre-eminent in furnishing facilities for the development of the most remarkable science ever investigated by man.

It would be pleasant to give some further detail of the history of the microscope in America, and refer to Drs. Bailey, Gillman, *Burnett and others, who have distinguished themselves as microscopists, but time forbids. At some future time this shall form a conspicuous part of another paper.

The microscope is beginning to be recognized as indispensable to every department of scientific research.

It is valuable to the naturalist in enabling him to solve that long debated question as to the vegetable origin of coal, as un-

* Dr. Burnett, of Boston, has since deceased, universally beloved and lamented. By his death, the cause of science has lost one of her most brilliant and successful votaries. Although taken away in his comparative youth, he has left behind him a name which will ever be distinguished in the annals of microscopical science.

der the microscope, its character is at once determined. From the smallest fragment of a fossil bone, you may with the microscope, aided by comparative anatomy, determine the form, structure, character and appetite of the animal to whom it belonged.

To the geologist, it determines the species of all fossils, and with certainty of all crystals, determines with accuracy, the constituents and character of all rock formations, by resolving apparently amorphous masses into their distinct characteristic forms, resolves the most compact granite into its ultimate constituents, crystals of quartz, mica and feldspar, &c., &c.

To the chemist, it unfolds the character of minute portions of chemical substances by their crystalline structure, and enables him to determine the angle of such crystals. It is of value also in witnessing the process of those minute chemical operations of nature, which involves the transposition of particles of matter invisible to the naked eye.

In entomology, it opens to us the beauties of insect life; the butterfly tinted like the rainbow, and exhibiting in its structure, the most elaborate mechanism, even to the remotest particles. Objects of disgust expand into a world of surpassing beauty; the caterpillar's skin exhibits the most gorgeous appearances and may be compared to the richest carpeting or tapestry; showing us that the eyes of insects are each composed of myriads of lenses, each of which is as perfectly formed as the human eye, each carrying their impression to a common retina; developing minute scales from their bodies, so distinctly and characteristically that even a fragment of one of these will enable us to determine with certainty the form and habits of the insect.

In botany, it introduces us to the theory of growth and enables us to descend into the secret recesses of nature to her innermost laboratories, there to witness and understand the theory and practice of her operations; to actually see the absolute progression of the formative process, as distinctly move on and build up as surely and progressively as a structure of art, under the hand of combined labor. To a great extent, it enables us to classify vegetable forms, point out the minute cellular structure of the most delicate flowers. In vegetable anatomy it determines whether the formation is exogenous or endogenous, increasing from within or without, and of fossil wood, whether it is coniferous or otherwise.

In the arts generally, it is of indispensable value to the metallurgist in determining the crystalline structure of various metals; for intance, the different qualities of iron ore, characterized by different conditions in crystallization, which can only be distinguished by the aid of the microscope. It detects the intermingling of different metals. In the examination of mixed metals which do not form strict chemical alloys, it enables you to detect the uncombined particles.

It is of great value in the examination of manufactured texture, in enabling us to determine the character of the fibre of which it is composed, guarding us against imposition. It is useful, too, in this respect in numerous other departments of art.

To the antiquarian, it opens the sealed volume of ages gone by, by disclosing those minute characters, ornaments and hieroglyphics, beyond the reach of ordinary vision ; it unfolds to him much of the history of the past, and, as in the case of the Egyptian antiquities already referred to, it even informs us of the existence of the microscope itself.

To the dental and medical student and practitioner, however, the microscope especially recommends itself. A new branch of medical and anatomical study, that of histology, has been created by its means alone, while its contributions to morbid anatomy, physiology and odontography, make it indispensable as an educational instrument to the student or practitioner who would excel, or even keep pace with the progress of others in his profession. To such the following remarks will be of interest: "histology, is that science which treats of the minute and ultimate structure and composition of the different textures of organized bodies.

The attempts of early microscopic observers to determine the ultimate structures, were in general of little value, partly on account of the imperfections in the instruments employed, and partly from the mistakes they made in the novel appearances presented to their view. This last cause still exists, and inexperienced observers may very readily be led astray.

Malpighi was the first to witness the most beautiful sight which the microscope can reveal, the actual circulation of the blood, thereby demonstrating the reasoning of Harvey to be true.

In investigations of the *teeth*, it not only exposes to our view their tubular and cellular structure, but it enables us to determine the character and habits of its owner, whether bird, fish, quadruped, biped or human, whether it ever gave its possessor pain, and if so, to a great extent the nature of the disease. It enables us too, with considerable accuracy, to determine the age, if human, of the individual whom it served.

It introduces us to a clear understanding of the formative process of nature in developing a tooth from the thinnest fluid pulp, to systematic form and flint, like density.

The broad field of undiscovered truth is before us, it is as wide and unlimited, and its supplies are as exhaustless as ever. It is full of beauty, abounding in the manifestations of wisdom and rich with the treasures of valuable and practical truth; knowledge and truth awaiting to bless mankind, and make its discoverer another benefactor of our race. Who shall enter this field of pleasing research? There are laurels, green laurels, hanging up in the temple of fame, impatient for the hour when they shall press the brow of him who shall prove himself worthy.

Let us all enter the arena, all will meet with a reward, and some will have an abundant one.

England is far in advance of our profession in this department of our art. She had her Nasmyth, peace to his memory. She has her Owen, her Goodsir and her Tomes, great and noble names; honor to them all! America, with the best artist, and the best microscope in the world, should do something more for our favorite science. In all other departments of our art, we are in advance of every other land, but here, with unrivalled facilities, we are far behind. Let us redeem ourselves.

